# A Transcriptome—Targeting EcoChip for Assessing Functional Mycodiversity

**DOI:** 10.3390/microarrays1010025

**Published:** 2011-10-31

**Authors:** Derek Peršoh, Alfons R. Weig, Gerhard Rambold

**Affiliations:** 1 Mycology, University of Bayreuth, 95440 Bayreuth, Germany; Email: derek.persoh@uni-bayreuth.de (D.P); Email: gerhard.rambold@uni-bayreuth.de (G.R); 2 DNA-Analytics and Ecoinformatics, University of Bayreuth, 95440 Bayreuth, Germany

**Keywords:** active fungal community, soil, quantitative microarray, precursor rRNA, mRNA

## Abstract

A functional biodiversity microarray (EcoChip) prototype has been developed to facilitate the analysis of fungal communities in environmental samples with broad functional and phylogenetic coverage and to enable the incorporation of nucleic acid sequence data as they become available from large-scale (next generation) sequencing projects. A dual probe set (DPS) was designed to detect a) functional enzyme transcripts at conserved protein sites and b) phylogenetic barcoding transcripts at ITS regions present in precursor rRNA. Deviating from the concept of GeoChip-type microarrays, the presented EcoChip microarray phylogenetic information was obtained using a dedicated set of barcoding microarray probes, whereas functional gene expression was analyzed by conserved domain-specific probes. By unlinking these two target groups, the shortage of broad sequence information of functional enzyme-coding genes in environmental communities became less important. The novel EcoChip microarray could be successfully applied to identify specific degradation activities in environmental samples at considerably high phylogenetic resolution. Reproducible and unbiased microarray signals could be obtained with chemically labeled total RNA preparations, thus avoiding the use of enzymatic labeling steps. ITS precursor rRNA was detected for the first time in a microarray experiment, which confirms the applicability of the EcoChip concept to selectively quantify the transcriptionally active part of fungal communities at high phylogenetic resolution. In addition, the chosen microarray platform facilitates the conducting of experiments with high sample throughput in almost any molecular biology laboratory.

## 1. Introduction

Microbial and fungal biodiversity analysis depends to a great extent on sequence-based identification of multipartite organismic associations or communities. Barcoding regions, such as rRNA and cytochrome oxidase loci, are currently used to characterize samples or isolates. In addition, corresponding community functions are inferred by analyzing the key enzymes of major processes like biomass production, nutrient cycling, and degradation of organic matter.

The number of published DNA and RNA sequences for phylogenetic affiliation and metabolic function has increased enormously over the past 15 years due to better availability of automated sequencing devices based on capillary electrophoresis. With the advent of so-called “next-generation sequencing technologies” (NGS; for an overview, see [1]), the identification of genotypes/taxa and functions has been greatly extended, not only towards obtaining information on underrepresented organisms (“rare biospheres”), but also towards the detection of organisms recalcitrant to cultivation. A recent re-examination of publicly available fungal sequences revealed that the vast majority of sequences corresponded to ribosomal RNA sequences (SSU, LSU, and ITS; [2]).

The benefit of NGS technologies is clearly evident for various applications such as amplicon sequencing of specific marker loci (e.g., [3,4,5]), expression profiling (e.g., [6,7]), or the detection of genomic variation within specific organisms (e.g., [8,9]). The fact that single environmental samples, especially from soil, may contain several thousands of microbial genotypes is, however, still the major reason why de novo genome assemblies from such samples have not yet been achieved. Similarly, full transcriptome analyses of complex environmental samples by sequencing cDNA cannot yet resolve the transcriptome of each taxon in the sample [10], but is restricted to comparative studies on selected genotypes or taxa. Hitherto, the major obstacle for biodiversity analyses of NGS transcriptome sequences from environmental samples has been the difficulty or impossibility of directly assigning individual sequences or contigs to the corresponding taxa or genotypes without the help of reference sequences or whole genomes. In addition, misassignments of artificial chimera sequences (sequence reads derived from more than one species assembled in a single contig) are unavoidable [11].

Pyrosequencing allows for generating relatively long reads (>500 bp) and is currently the preferred technique for amplicon sequencing, e.g., barcoding loci. In recent studies, it exhibited an enormous diversity of fungal genotypes in soil samples [12] as well as a strikingly high bacterial diversity, for instance, in deep sea and oral microflora samples [13,14]. The reliability of such biodiversity data or estimates, however, has been questioned by the results of community simulation modelling [15]; based on the observation that the application of NGS technologies puts up with high sequencing error rates, the presence of singleton sequences has been mainly ascribed to artefacts rather than to real but underrepresented operational taxonomic units.

With respect to the apparent lack of broad functional enzyme sequence coverage in fungi, but considerably rich phylogenetic (barcoding) sequence information in these organisms, we explored a new experimental strategy to use sequence data for the quantitative analysis of microbial soil communities and to measure community composition and functions based on public sequence data.

This novel approach is based on simultaneous but independent analyses of functional and phylogenetic hybridization signals in the same microarray experiment. For this “dual-probe-set” (DPS) approach (Table 1), microarray probes for functional enzymes were designed from conserved domains of enzymes, which may not necessarily contain phylogenetic information. Targeting these regions of low sequence variability should not only allow for the detection of functional transcripts by perfect match hybridization, but also of transcripts from unidentified organisms by cross-hybridization. The loss of phylogenetic information of those functional enzyme probes is compensated for by additional microarray probes designed from internal transcribed spacer sequences (ITS) of rRNA genes, which are highly variable fungal barcoding genes. These transcripts are present in eukaryotic cells as short-lived 45S-precursor rRNAs in amounts comparable to specific mRNA species, and, thus, should allow simultaneous quantification of mRNA and rRNA transcripts.

**Table 1:** Comparison of different target sites in rDNA and functional enzyme sequences: probes targeting the internal transcribed spacer sequences (ITS) regions of rRNA and conserved domains in functional enzymes together constitute the dual probe set (DPS). The classical GeoChip approach is also indicated.

	rDNA repeat	Functional enzymes
Structural organization	SSU/5.8/LSU	ITS1/2	conserved domains (e.g., active sites, structural determinants)	less conserved regions
Complexity of metagenomes	low	high	low	high
Amounts of molecules in total RNA	high (as mature and precursor rRNA)	low (only as precursor rRNA)	between different genes)
resolution	medium to low	high	low	high
Probe set		↳ **DPS** ↲	“GeoChip”

While all available functional enzyme sequences of fungi were considered for the design of the pilot array, a selection was made for assessing the applicability of probes targeting the precursor rRNA. The assortment included all sequences from the “basal fungal lineages”, tagged Zygomycota in the following, and representatives of the Ascomycota (*i.e.*, Eurotiales) and Basidiomycota (*i.e.*, Agaricales). The chosen taxa reflect the ITS sequence variability among fungi and are regularly present in soil.

## 2. Results

**Figure 1:**
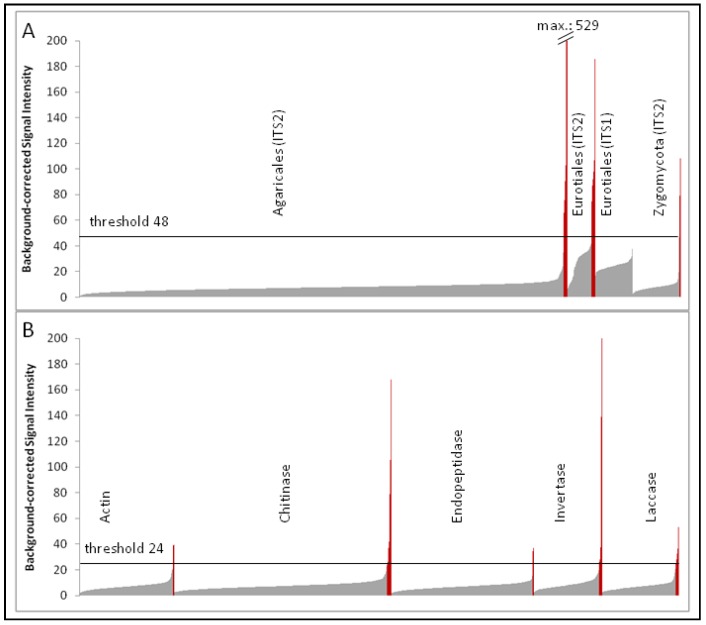
Signal intensity histogram of precursor-rRNA probes (A) and transcripts of genes encoding enzymes analyzed in this study (B); probes were grouped according to their original taxonomic assignment or according to their (putative) enzymatic activity and ordered by ascending signal intensity (taken from the Cy3 channel of the “Myk1” hybridization sample; see GEO accession GSE28018). Grey and red bars indicate probe signals below and above the threshold values of each target group, respectively.

The independent single-dye hybridization experiments (two labeled RNA samples hybridized at four different sample amounts to eight different subarrays) resulted in highly comparable signal intensities between the two corresponding samples. Although the strong signals could even be detected using a 125-fold diluted labeled RNA sample, the weaker signals were no longer recognizable. Therefore, probe signals obtained from the pair of hybridization experiments using undiluted labeled RNA were used for subsequent analysis (labeled as “Myk1” and “Myk2” in the NCBI Gene Expression Omnibus (GEO) accession GSE28018, respectively; see also experimental Section 4.6.). Based on a histogram of ascending signal intensities of the various probe groups (Figure 1), it was possible to distinguish the majority of unspecific signals close to the background value from the few specific probe signals. A threshold value of twice above the background (normalized values above 24 in the Cy3 channel) was selected to separate specific probe signals of enzyme transcripts from unspecific hybridization signals. A similar signal intensity profile was observed for precursor rRNA probes of Agaricales and Zygomycota, but not for Eurotiales probes. The majority of probe signals of the Eurotiales group exhibited significantly higher unspecific hybridization signals, which are most likely due to the comparatively smaller genetic distances among Eurotiales probe sequences compared to those among the Agaricales or Zygomycota. Therefore, a threshold value of four-fold above the background value (normalized values above 48 in the Cy3 channel) was applied to all ITS probe signals (including the Agaricales and Zygomycota probes). Correlation analysis of specific probe signals (above the threshold) revealed that signal intensities could be reproduced using undiluted labeled samples with an R2 value of 0.94 for precursor rRNAs.

### 2.1. Identification of Precursor rRNA Transcripts as Phylogenetic Markers for Fungal Soil Communities

Detailed post-experimental evaluation of ITS probe sequences exhibiting above-threshold hybridization signals (Figure 1A) revealed that only a fraction of these probe sequences really targeted members of the Agaricales, Eurotiales, or Zygomycota, respectively, indicating a serious misassignment of taxonomic information in public sequence databases (Table 2). Of the 48 strong ITS signals, only 13 signals indicated the presence of Agaricales (five signals) and Eurotiales (six signals). Two additional sequences indicated the presence of *Paecilomyces* (Eurotiales). However, these were similar to congeneric taxa that have recently been transferred to the Hypocreales [16]. The remaining 35 strong signals predominantly indicated the presence of Sordariomycetes, represented by Hypocreales (14 signals), Sordariales (two) and Xylariales (two), and the genus *Arthrinum*.

**Table 2:** ITS rRNA probes with signal intensities above the threshold. The signal intensities in the two experiments (Myk1 and Myk2, cf. GEO GSE28018) are given for each probe. The fourth column indicates the taxonomic group actually targeted by the probe, as revealed by a BLAST search. The number of sequences among the perfect matches, deposited under matching (matches), contradicting (outliers), or lower level (ambiguities) taxonomic names is noted in parentheses. The ordinal affiliation of the target group is given in the last column.

Probe ID	Signal intensity (Myk1)	Signal intensity (Myk2)	Target group(matches/outliers/ambiguities)	Order
ITS_01_50002900	107.7	156.4	*Cladosporium* (199/1/300)	
ITS_01_50005177	92.2	160.3	*Cladosporium* (200/1/299)
ITS_01_50001997	90.6	90.7	*Cenococcum* (89/0/255)	Dothideomycetes inc. sed.
ITS_01_50002490	140.7	221.3	Myxotrichaceae (8/2/6)
ITS_01_50005012	86.8	112.9	*Aspergillus* (1/0/0)	
ITS_01_50005015	52.1	92.1	*Aspergillus* (1/0/0)
ITS_01_50006139	49.2	78.1	*Aspergillus* (72/0/27)
ITS_01_50005104	185.6	248.2	*Aspergillus* (1/0/0)
ITS_01_50005004	104.2	174.0	*Paecilomyces carneus* (7/2/4) *
ITS_01_50005051	66.9	126.2	*Penicillium* (1/0/0)
ITS_01_50005142	74.8	106.9	*Penicillium* (1/0/0)
ITS_01_50005035	88.1	127.0	*Nalanthamala vermoesenii* (59/13/3) *	Eurotiales/Hypocreales
ITS_01_50005171	98.1	161.2	Cordycipitaceae (116/0/5)	
ITS_01_50005106	87.7	152.6	Cordycipitaceae/Ophiocordycipitaceae (167/2/23)
ITS_01_50002531	98.5	130.5	*Fusarium* (324/13/163)
ITS_01_50002532	100.5	149.8	*Fusarium* (321/4/50)
ITS_01_50005175	99.0	162.1	*Fusarium* (324/13/163)
ITS_01_50005053	108.7	189.5	*Fusarium* (277/6/122)
ITS_01_50005105	87.3	131.0	*Isaria* / *Paecilomyces* (40/2/1)
ITS_01_50005159	95.8	162.3	*Isaria cateniannulata* (1/0/0)
ITS_01_50005158	106.7	166.4	*Isaria cateniannulata*/Evlachovaea (26/2/3)	
ITS_01_50002492	102.7	175.2	*Isaria* (17/4/0)
ITS_01_50005170	97.8	181.4	*Isaria* (17/4/0)
ITS_01_50005041	81.5	111.5	*Nalanthamala psidii* (9/5/0)
ITS_01_50005176	95.1	139.8	*Torrubiella luteorostrata* (3/2/0)
ITS_01_50005161	90.8	160.3	*Torrubiella luteorostrata* (3/0/0)
ITS_01_50005153	81.0	158.6	*Leptosphaeria* (11/6/40)	
ITS_01_50001996	66.1	97.3	Pleosporaceae (259/2/239)
ITS_01_50006165	108.5	147.2	Pleosporaceae (260/4/236)
ITS_01_50001499	75.7	120.6	Pleosporales (302/7/191)
ITS_01_50006164	78.5	121.9	Pleosporales (286/10/179)
ITS_01_50006166	79.0	108.2	Pleosporales (306/8/186)
ITS_01_50001886	106.3	179.1	Chaetomiaceae/Lasiosphaeriaceae (69/1/121)	
ITS_01_50005102	98.9	158.6	Chaetomiaceae/Lasiosphaeriaceae (69/2/120)
ITS_01_50003051	67.5	141.6	*Arthrinium* (96/6/16)	Sordariomycetes inc. sed.
ITS_01_50003052	66.0	147.5	*Pestalotiopsis* (88/4/7)	
ITS_01_50005179	60.2	103.8	*Pestalotiopsis* (89/3/7)
ITS_01_50000693	529.3	516.2	*Amanita constricta* (1/0/0)	
ITS_01_50000694	497.0	600.8	*Amanita constricta* (2/0/2)
ITS_01_50000692	479.4	576.5	*Amanita liquii* (2/0/0)
ITS_01_50000709	92.5	176.3	*Amanita virosa* (1/0/0)
ITS_01_50002501	60.9	84.7	*Hygrocybe* (1/0/0)
ITS_01_50002530	83.5	88.0	*Lactarius* (9/1/2)	
ITS_01_50001012	90.0	88.7	*Russula* (3/0/0)
ITS_01_50000049	69.7	64.0	Russulaceae (1/0/0)
ITS_01_50001973	83.2	83.1	Russulaceae (1/0/0)
ITS_01_50001863	67.8	116.9	Mycota (9/0/0)	
ITS_01_50006167	73.7	111.7	Mycota (1/0/2)
* Two probe sequences targeting species of Eurotiales clustered among the Sordariomycetes-specific sequences, one matching *Paecilomyces carneus*, the other *Paecilomyces lilacinus* (Eurotiales) and *Nalanthamala vermoesenii* (Hypocreales, Sordariomycetes). As several of the anamorphic *Paecilomyces* spp. were shown to correspond to Hypocrealean teleomorphs [16], these two *Paecilomyces* spp. likely represent Hypocrealean anamorphs as well.

### 2.2. Identification of Microbial Community Function using Conserved Enzyme Domains

Microarray probes designed to integrate transcripts of different taxa via conserved protein domains displayed several hybridization signals above the threshold value (Figure 1B). Although these functional probes were designed to cover broad sequence variation at conserved protein sites, only a small fraction of probes was actually labeled by RNA from soil samples. Of the three domains selected for chitinase probe design (508, 499, and 502 probes, respectively), 10 hybridization signals were detected with probes targeting the first domain and seven signals were obtained with probes targeting the third domain. The second targeted sequence domain showed no hybridization signals above threshold values.

Two conserved regions were selected for the design of 501 and 490 endopeptidase probes, respectively. The hybridization results revealed that significant signals were found with three probes designed from the first of the two conserved endopeptidase regions.

Laccase probes were designed from two conserved domains (254 and 280 probes, respectively); laccase transcripts were detected by three probes, two of which targeted site 1.

The amount of plant-derived RNAs within the total RNA samples from soil seemed to be comparably small, since only 5 and 17 probes encoding plant actin and invertase transcripts exhibited signals above the exclusion limit, respectively. Again, these sequences were designed to conserved domains in plant actin and invertase protein sequences and were used to estimate the amount of plant RNA within the total RNA preparation of the soil sample.

**Figure 2:**
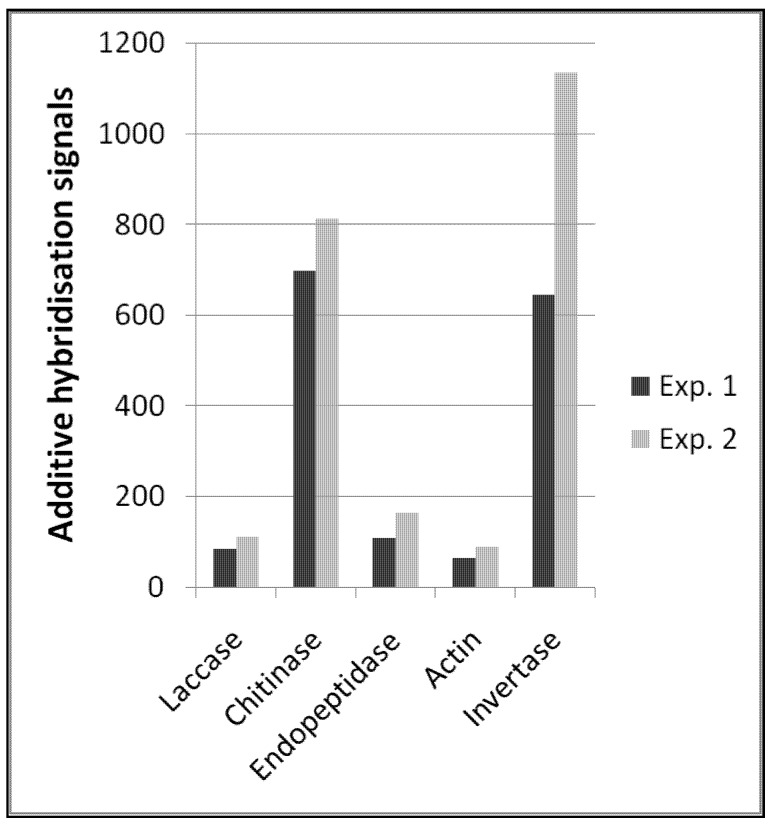
Expression profile of selected functional enzymes obtained from two independent hybridisation experiments; for detailed probe description, see the Experimental Section.

In summary, the functional enzyme signals allowed us to quantitatively describe transcript amounts of different functional enzymes (Figure 2). Reproducibility between these types of probes was also high between the two independent experiments, which is a prerequisite for comparative quantitative analyses. Interestingly, the amount of plant invertase transcripts was considerably higher and less reproducible. However, the spatial distribution and amount of plant tissues (e.g., roots) in soil samples is certainly not comparable to the distribution of fungal microorganisms, and one can expect that the presence or absence of such macroscopic tissues like roots will cause greater variability in hybridization signals.

## 3. Discussion

A diagnostic microarray to advance functional biodiversity research of fungal communities from environmental samples is expected to fulfill two requirements: first, to exhibit broad taxonomic and functional coverage with high diagnostic specificity, and second, to be open to additional molecular markers and functional groups as they will henceforward become available. Although quite a number of genes encoding enzymes involved in environmental function are already known from fungi, available markers used in phylogeny, such as rRNA gene sequences, massively exceed in number those of protein coding sequences [2]. To bridge the apparent disequilibrium of marker resolution, a new microarray design approach was evaluated on a high-density microarray (Table 1).

The results of the novel EcoChip approach showed that distinct positive signals were simultaneously detected for the precursor ITS probes and the probes targeting conserved protein domains (both constituting the DPS) which were readily distinguishable from unspecific cross-hybridization signals. Hybridization specificity could be also confirmed using an additional set of control probes designed in a traditional way targeting selected fungal endopeptidase sequences (data not shown). The applicability of the microarray approach for functional biodiversity analysis could therefore be confirmed in principle.

The finding that precursor rRNA is present in such amounts that ITS probes yielded considerable signal intensities allows for selectively detecting vital (transcriptionally active) organisms in microarray experiments, exclusive of dormant and dead fungi. All taxa detected by the ITS probes (Table 2) are known to be soil fungi, which confirms the coherence of the results from a biological perspective. Detailed inspection of the data revealed some shortcomings of the pilot microarray and several starting points for the optimization of future experiments, which are discussed in detail in the following section.

### 3.1. Probe Design

The considerably smaller number of significant hybridization signals by conserved-domain targeted probes can be explained by the nature of these probes, which integrate transcripts of several taxa under a few functional probes. The amount of functional enzyme transcript signals should therefore be considered as a measure of the overall functional activity within a (soil) sample. While the more abundant taxa probably contribute more to the functional transcript signals, the presence of highly specialized but rare fungi with elevated functional enzyme expression cannot be excluded. The assignment of functions to taxa therefore requires correlation analyses of functional and phylogenetic data from a series of EcoChip experiments, as usually conducted in ecological studies.

Re-analysis of the manually selected conserved-domain targeting probes using probe design software (AlleleID, Premier Biosoft International) revealed that the overall “probe quality” value (a measure calculated by AlleleID) of conserved-domain targeted probes was significantly lower compared to probes designed by the software itself (e.g., the endopeptidase control probe set; data not shown). Advanced probe design strategies (e.g., the cross-species probe design feature available in AlleleID) should henceforth also be used for obtaining probes targeted to conserved regions and could result in elevated hybridization signals.

### 3.2. Taxonomic Sequence Annotation

**Figure 3:**
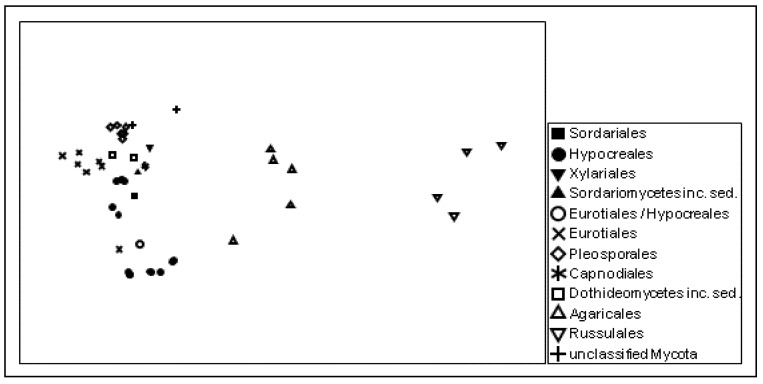
Similarity among the ITS rRNA probe sequences giving positive signals, as visualized by non-metric multidimensional scaling (NMDS). The taxonomic affiliation of the detected groups is indicated at the order level; stress: 0.1.

Although the large amount of sequence information available from traditional approaches and next-generation sequencing projects allows for designing phylogenetic microarrays at various phylogenetic levels, the taxonomic annotation of high throughput sequence data is an issue which must be seriously addressed. Our results confirmed previous results [17] showing that a considerable number of published sequences are obviously misannotated. Individual sequence comparison of the probes with publicly available sequence databases revealed that the majority of the detected putative Agaricales, Eurotiales, and Zygomycota sequences need to be assigned to totally different fungal orders. This finding became obvious from the multidimensional scaling results of the genetic distances of the probe sequences, which reflect the actual phylogenetic relationships among the targeted taxa very well (Figure 3).

Evaluation of the ITS probe sequences against the background of published sequences also revealed certain limitations in taxonomic resolution (Table 2). A few sequences were characteristic for certain species, but most signals just indicated the presence of a certain genus. While shifting the precursor rRNA target sites towards more variable sites of the ITS regions may increase taxonomic resolution, even these highly variable regions are insufficient to discriminate between all fungal species. However, the generic affiliation is certainly sufficient for addressing most ecological questions. Multi-gene phylogenies, as required for species delimitation in several fungal taxa, are not feasible with DNA/RNA extractions from environmental samples and require isolation of the organisms of interest in pure culture.

The currently used workflows for reliable sequence annotation [18,19] are rather time-consuming and an automated approach is desirable. Since cross-hybridization of closely related sequences cannot be completely avoided in microarray hybridization experiments, assignment of hybridization signals to specific taxa should also account for the limit of phylogenetic resolution of such experiments. For an efficient evaluation of high density microarrays, a phylogenetic assignment algorithm including experimentally quantified cross-hybridization events of various mismatch probes is therefore indispensable.

Accounting for cross-hybridization would reduce the phylogenetic resolution by adding closely related taxa to the list of organisms targeted by phylogenetic probes. The resulting decrease in phylogenetic resolution may be compensated for by shifting the precursor rRNA target sites towards more variable sites of the ITS regions. However, the contribution of cross-hybridization to the overall hybridization signal according to the genetic distance of the considered taxa has not yet been explored. The evaluation applied in the present context is therefore solely based on the assignment of perfectly matching sequences.

### 3.3. Cross-Hybridization versus Specificity

Two major aspects of functional biodiversity research, the analysis of community composition (presence of taxa in a sample) and the analysis of biological functions (processes triggered by community members), were addressed by the pilot microarray. Here, function and taxonomy have to be detected by independent probe sets (the DPS approach), with contrary demands on cross-hybridization. With respect to conserved amino acid sequence domains of functional genes, short stretches of considerably high nucleic acid sequence similarity can be targeted by a limited number of microarray probes. As a consequence, conserved-domain targeting probes can comprise more taxa under one probe signal by cross-hybridization, even in the case that a limited number of reference sequences are available. At the same time, cross-hybridization should be minimized for ITS probes to increase phylogenetic resolution. The observed elevated background signals for the Eurotiales targeting ITS probes indicated greater cross-hybridization due to lower sequence variability compared to the Agaricales and Zygomycota probe sets. Indeed, each Eurotiales probe matched 2.5 taxa on average (179 probes recognized 460 taxa), indicating the presence of different taxa sharing identical sequences. In contrast, each Agaricales and Zygomycota probe matched about 0.7 taxa within these groups, indicating the presence of taxa with considerable sequence polymorphisms (“intraspecific variation”). These values are calculated for a perfect match and will increase if sequences with a certain number of mismatches (cross-hybridization) are also accounted for during data evaluation. To determine a threshold for the search of matching sequences by evaluating positive signals from phylogenetic probes, detailed cross-hybridization experiments with synthetic oligonucleotides are necessary. ITS regions are, in principle, large and diverse enough to design specific probes for optimal phylogenetic resolution. Therefore, additional target sites within ITS regions may be chosen with the consideration of such experimental results for future optimized EcoChip arrays. Such experiments will also allow for a theoretical assessment and elimination of signals due to cross-hybridization between similar probe sequences.

### 3.4. Probe Accessibility of rRNA Sites

It has already been shown in several studies (see below), that a specific probe signal intensity depends not only on the amount of the particular nucleic acid species (e.g., transcripts) in a sample, but also on the kind and degree of secondary structure of the hybridizing regions, which may greatly influence the hybridization of labeled samples to the microarray probes. While the effect of secondary structure has been studied in detail for mature rRNA (e.g., [20,21,22]), equivalent studies on ITS regions are still missing, although detailed secondary structural information is already available at least for the ITS2 region [23]. Probe accessibility issues, however, also apply to mRNA genes [24]. Our results obtained from at least two independent probe sequences for each transcript revealed significant differences in signal intensities, which, however, could not be explained by secondary structure alone. This is in accordance with studies on the rRNA accessibility of FISH probes, which cannot be satisfactorily predicted *in silico* and must be experimentally tested (cf. [22]). Actually, it appears most reasonable to apply multiple probes [25] designed by advanced algorithms [26,27] for each target sequence. In the long run, cumulative accessions in probe sequence databases like probeBase [28] will certainly facilitate predictions on the accessibility of phylogenetic and functional markers for fungi and other eukaryotic microorganisms.

### 3.5. Interplay of Next-Generation Sequencing and Next-Generation Functional Biodiversity Microarrays

The outcome of the pilot microarray experiments is promising in that NGS sequence data (like any other sequence data) can be easily incorporated into a robust quantification platform. Our results strongly support the opinion of Roh and co-workers [29] who considered the complementary use of microarray and NGS technologies in the field of microbial ecology. In this context, we suggest modifying and extending the notable features of DNA microarrays (Table 1 in [29]) considering the following aspects: (1) The preparation of microarray slides no longer needs to be in the hands of the researcher but is carried out via the provider’s manufacturing pipeline, ensuring the production of high-quality microarray slides. In addition, the considerable high oligonucleotide synthesis costs of conventional (spotted) microarrays no longer apply to in situ synthesis processes. (2) The well-established expression analysis workflow for this type of microarray enables hybridization experiments to be performed in almost any laboratory with access to microarray scanners and common molecular biology equipment. (3) The use of chemical labeling techniques of total RNA probes does not require amplification steps which are prone to introducing artefacts or biases which make quantitative estimates difficult.

We expect that a sophisticated combination of NGS and “next generation microarray” (such as EcoChip) techniques has great potential to substantially support environmental research both at the qualitative and quantitative levels. And, as serious issues have been raised with regard to next-generation sequence data quality [15], we think now is the appropriate time to suggest additional experimental procedures for using such data in the context of functional biodiversity research as well.

## 4. Experimental Section

### 4.1. Microarray Design

For establishing the RNA EcoChip, the 8 ✕ 15 k microarray layout (Agilent Technologies) was chosen. This allowed the placement of eight identical subarrays on a single slide. Each subarray consisted of 15,744 spots, whereof 536 spots were reserved for Agilent control probes. Due to the finding that the solution-facing 5’ half of a 60-mer oligonucleotide almost exclusively determines the hybridization specificity, while sequence variations of the 3’ half of an oligonucleotide probe (the end attached to the slide surface) hardly affects probe specificity [30], all microarray probes were initially designed as short probe sequences of about 30 bp and, in a second step, the short probes were extended at the 3’ end to a final length of 60 bp using the adjacent sequence of each target sequence.

### 4.2. DPS Probe Design from ITS Sequences

Precursor rRNA probes were deduced from about 15,000 database accessions available at the NCBI sequence repository [31] in April 2010. In contrast to the identification of enzyme coding sequences, the database query was limited to sequences assigned to Eurotiales (3980 ITS sequences), Agaricales (9929), and “basal fungal lineages” (2009, tagged “Zygomycota” in the following), respectively. Representatives of these groups are known to frequently dominate soil fungal communities [32]. The partial multiple sequence alignment for each group was restricted to the conserved SSU, 5.8S and LSU rRNA sequences, to locate the border between the mature rRNA and the spliced ITS regions of the precursor rRNA. All probes were designed from the opposite strand of the ITS regions adjacent to the 5.8/ITS2 border and the SSU-ITS1 border, respectively, with an initial length of 30 bp. These probes were then extended at the 3’ end into the 5.8S and SSU regions, respectively, to a final length of 60 bp (see above). From 3980 sequences (representing about 460 taxa of Eurotiales) available at NCBI in April 2010, 179 unique probe sequences were designed for the 5.8S-ITS2 border and 243 for the SSU-ITS1 border. The 9929 sequences of Agaricales (ca. 2250 taxa) were targeted by 3137 probes at the 5.8S-ITS2 border. The 2009 sequences of Zygomycota (ca. 200 taxa) were covered by 306 unique probes targeting the 5.8S-ITS2 border.

### 4.3. DPS Probe Design from Enzyme Coding Genes

Functional transcript probes for the DPS approach were designed for (putative) chitinases, endopeptidases, and laccases from fungi and for actin and invertase proteins from plants. Amino acid sequence alignments from all available fungal sequences were used to identify conserved sequence domains and probe sequences were deduced from at least two of these highly conserved domains. This approach was intended to increase the probability that microarray probes would also detect transcript sequences of phylogenetically related, but not yet identified (and sequenced) soil microorganisms (alignment files consisting of all sequences used for conserved domain detection are available upon request). Again, a first probe sequence targeted 30 bp in the highly conserved domain, which was then extended to a final length of 60 bp. Three conserved regions were targeted by the 1107 available fungal chitinase mRNAs from 245 taxa. The sequence diversity at site 1 was covered by 508 unique probes (probe IDs Chi_01_11000002 – Chi_01_11000509), at site 2 by 499 probes (Chi_02_11100001 – Chi_02_11100499), and at site 3 by 502 probes (Chi_03_11200001 – Chi_03_11200502). These sites correspond to the amino acid sequences GFDGIDLDWEFPGNNESEPR, ITEIDQYVDYWNMMTYDYYG, and KLVLGMAAYGRSFHIKDNKF of NP_010659 (*Saccharomyces cerevisiae*), respectively. Two conserved regions of the 1076 endopeptidase sequences from 51 taxa were targeted by 501 (site 1: probe IDs Epa_01_12000001 – Epa_01_12000501) and 490 probes (site 2: Epa_02_12100001 – Epa_02_12110490), respectively. These sites correspond to the amino acid sequences TPSQSLTVLFDTGSADFWVM and PVLLDSGTSLLNAPKVIADK of NP_012249 (*S. cerevisiae*), respectively. The 756 laccase mRNAs were also targeted to two conserved sites, with 254 (site 1: Lcc_01_10000001 – Lcc_01_10000254) and 280 probes (site 2: Lcc_02_ 10100001 – Lcc_02_ 10100280), respectively. These sites correspond to the amino acid sequences VHVNNHLEEGQSIHWHGLR and SFTYQFTVSHQSGTFWWHS of ABI58272 (*Cryptococcus neoformans*), respectively.

### 4.4. Control Probe Design, Custom Microarray Production and Data Availability

The remaining probe positions on the microarray were reserved for internal controls such as homopolymers and other phylogenetic detection markers such as mature rRNA targets. In addition, another set of specific control probes targeting endopetidase-encoding transcripts were designed for evaluation purposes, using a well-established probe design tool (AlleleID ver. 7.50, Premier Biosoft International). The microarray was ordered via Agilent’s eArray web portal [33]. The probe sequences used in this work as well as the normalized hybridization data are available at NCBI’s Gene Expression Omnibus [34] under accession number GSE28018.

### 4.5. Sample Preparation and Labeling

Total RNA was isolated according to [35] from 0.4 g of the organic layer of an acidic podsol from a mixed forest site with predominant *Pinus sylvestris* L. on the hill “Hohe Warte” near the city of Bayreuth, Germany (49°58'16'' N, 11°34'51'' E, 460 m alt.; sampled at June 3, 2010). Next, 115 μmol of Al2(SO4)3 were applied to quantitatively flocculate the humic substances prior to cell disruption. The primary RNA samples were treated with DNase I and purified. Each 1.5 µg sample of RNA from two RNA preparations were labeled with Cy3 and Cy5 using the CyScribe Direct mRNA Labeling Kit (GE Healthcare); labeled RNA was fragmented (fragmentation reagent, Applied Biosystems/Ambion) prior to the hybridization experiment.

### 4.6. Microarray Hybridization and Signal Quantification

For each of the two soil samples, four independent hybridizations were carried out using different amounts of Cy3- and Cy5-labelled total RNA: either 1 µl of undiluted labeled and fragmented RNA (ca. 136 ng RNA; subarray Myk1 and Myk2) or water-diluted labeled RNA (ca. 27.2 ng, 5.44 ng, and 1.08 ng RNA per µl, corresponding to subarrays Myk3/4, Myk5/6, and Myk7/8, respectively) was combined with 2 µl 10X Blocking Agent and 25 µl 2X GEx Hybridization Buffer HI-RPM (both included in the Gene Expression Hybridization Kit, Agilent Technologies) in a total volume of 50 µl. Each hybridization mixture was added to one of the eight subarrays present on the microarray slide. The microarray slide was covered with a gasket slide and hybridization was carried out in a hybridization oven at 65 °C for 18 h. The microarray was washed using the Gene Expression Wash Buffer Kit (Agilent Technologies) as recommended. Stabilization and Drying Solution (Agilent Technologies) was added to the microarray slide to minimize ozone-induced Cy5 degradation. Microarray images were obtained using a microarray scanner (FLA 8000, Fuji) at a scanning resolution of 5 µm. Microarray signals were quantified using ArrayVision ver. 8.0 (GE Healthcare). The following key settings were used: (a) artefact-removed volume as the principal measure, (b) mean background values calculated from the surrounding region of each subarray, and (c) spot segmentation to compensate for minor differences in spot morphology.

Data normalization was carried out in two steps: first, local background values were subtracted from the raw signal data to obtain background-corrected hybridization signals; second, the ratio (Cy5/Cy3) of Eukarya-specific small subunit (SSU) rRNA signals (mean of four replicate spots in each subarray) was calculated separately for each pair (same sample dilution) of hybridization experiments and was used to normalize the background-corrected hybridization signals of the Cy5 experiment. Microarray data presented in this manuscript were deposited at NCBI’s GEO database under record GSE28018 [34].

### 4.7. Signal Affiliation and Taxonomic Consistency

Sequences of probes with signal intensities well above local background intensities were matched against the sequences deposited at NCBI using “Mega BLAST” [36]. A consensus name was attributed to each probe based on the taxonomic affiliation of all perfect matching sequences, as described previously [18]. The number of sequences deposited under a name matching the consensus name (“matches”), disagreeing with it (“outliers”), and having been assigned to a lower phylogenetic level (“ambiguities”) were assessed according to the taxonomy of Index Fungorum [37] and noted for each probe sequence giving a positive signal (Table 2). In addition, for probes matching only up to three sequences, the taxonomic affiliations of the target sequences were also checked.

For visualization, the similarity among the ITS probe sequences was determined by conducting a local BLAST search [38]. The resulting similarity values were transformed to a similarity matrix using the R script RFLPtools [39]. A non-metric multidimensional scaling (NMDS) analysis on the similarity matrix was created with Primer6 (ver. 6.1.11, Plymouth Routines).

## 5. Conclusions

Although high-throughput analysis technologies such as next generation sequencing will certainly be the major application for exploring biodiversity in different habitats, a quantitative analysis of ecosystem functions is still out of reach even for this sophisticated technology. This analytical gap can be complemented by the presented EcoChip concept and can be easily adapted to different functions by replacing the functional enzyme probe sets with those of other enzyme groups (leaving the phylogenetic probes unchanged). In addition, the comparably low cost of an EcoChip hybridization experiment offers a very attractive quantitative method when a series of environmental samples needs to be analyzed.
